# A technique to identify the axillary nerve and its cutaneous branch for triceps nerve-to-deltoid nerve transfer. A case report

**DOI:** 10.1016/j.ijscr.2018.11.023

**Published:** 2018-11-17

**Authors:** Mohammad M. Al-Qattan, Doaa Andejani, Ahmed Thallaj

**Affiliations:** a Division of Plastic and Hand Surgery, Department of Surgery, King Saud University Riyadh, Saudi Arabia; b Department of Anesthesia, King Saud University Riyadh, Saudi Arabia

**Keywords:** Technique, Identify, Axillary nerve, Triceps nerve-to-deltoid nerve transfer

## Abstract

**Introduction:**

Triceps nerve-to-deltoid nerve transfer requires the identification of the axillary nerve in the quadrilateral space. This may be difficult especially for residents-in-training. The senior author is a Professor of Hand Surgery at a teaching institution and has devised a new technique of identification of the axillary nerve and its cutaneous branch using surface land-marks and on-table ultrasonography.

**Case report:**

The axillary nerve and the quadrilateral space is first identified using anatomical landmarks. The ultrasound probe is the used to identify the quadrilateral space and the axillary nerve which appears as hyper-echoic oval-shaped structure. The ultrasound probe is then moved to scan the cutaneous branch of the axillary nerve as it branches-off the main nerve trunk. Finally, the cutaneous branch is traced superficially till it becomes subcutaneous. This point is marked and the skin incision is made along this marked point. During surgery, the cutaneous branch is followed retrograde to the axillary nerve in the quadrilateral space.

**Discussion:**

Ultrasound guidance for localization of various nerves is now routinely done by anesthetists in the Operating Room. This localization is used for nerve blocks and in patients with brachial plexus injuries. Hence, the ultrasound machine and the expertise are already available in the operating room; and no special arrangements with the Radiology Department are needed.

**Conclusion:**

A technique of identification of the cutaneous branch of the axillary nerve using anatomical landmarks and ultrasonography is described. The localization is accurate and is of help in patients undergoing triceps nerve-to-deltoid nerve transfer.

## Highlights

• Triceps nerve-to-deltoid nerve transfer requires the identification of the axillary nerve.
• We describe a new technique of identification of the axillary nerve.
• Anatomical landmarks are used first.
• The ultrasound probe then identifies the cutaneous branch of axillary nerve.
• During surgery, the cutaneous branch is followed to the axillary nerve.

## 1 Introduction

Triceps nerve-to-deltoid nerve transfer has become the standard-of-care in patients with isolated axillary nerve injury, isolated C5-C6 root avulsion of the brachial plexus, and in salvage of the paralyzed deltoid muscle following unsatisfactory intra-plexus neurotization of the posterior division of the upper trunk of the brachial plexus [[1], [2], [3]].

Identification of the axillary nerve in the quadrilateral space may be difficult especially for residents-in-training. The senior author (MMA) is a Professor of Hand Surgery at a teaching institution and has devised a new technique of identification of the axillary nerve and its cutaneous branch using surface land-marks and on-table ultrasonography. The technique may also be helpful for experienced surgeons in difficult cases such as obese patients and in case who had previous surgery in the arm. We describe the technique using demonstrative case. The work has been reported in line with SCARE criteria [4].

## 2 Case presentation/description and the technique

**Fig. 1:**
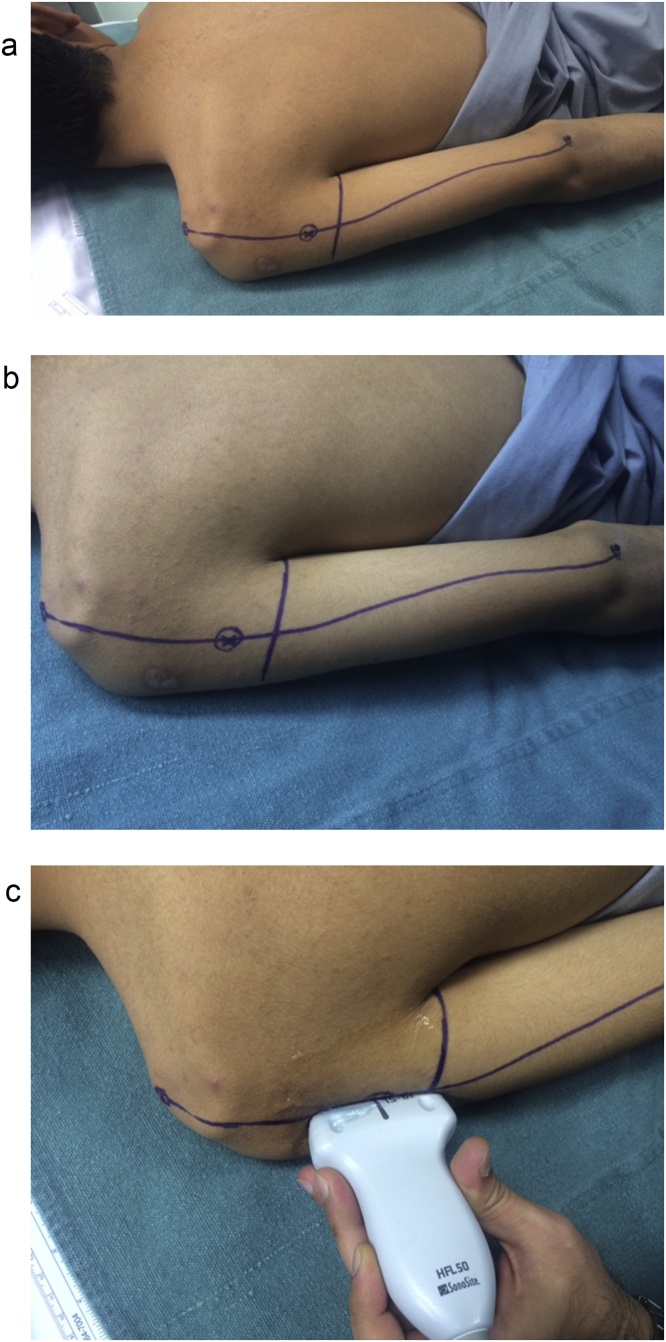
a) The longitudinal acromion – olecranon line transecting the transverse line from the axillary fold. Anatomically, the quadrilateral space is 2 cm cranial to this transection point. b) A close up of the two lines and the point (x) localizing the quadrilateral space. c) The ultrasound probe is use.

**Fig. 2:**
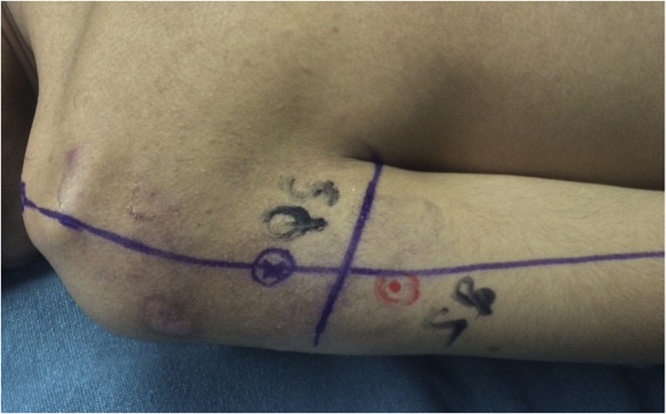
The red circle marks the localized cutaneous branch of the axillary nerve at the skin level (located by ultrasound).

**Fig. 3:**
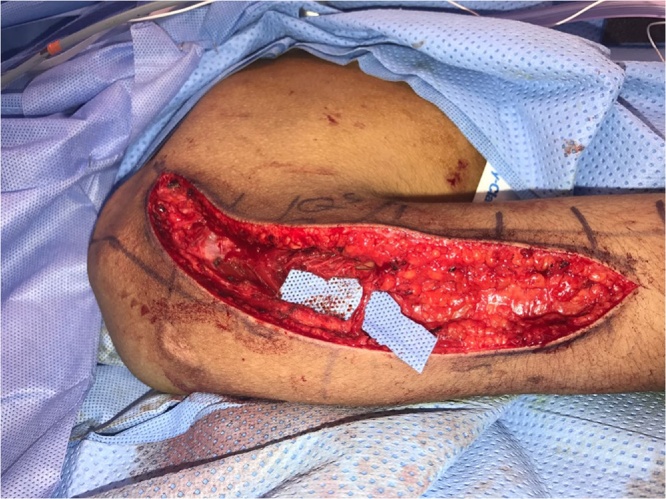
The cutaneous branch of the axillary nerve is dissected (shown under blue back-ground).

**Fig. 4:**
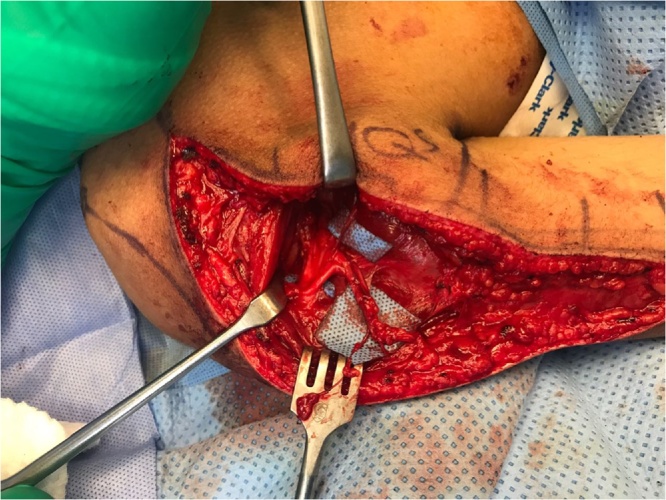
The cutaneous branch is traced retrograde to the axillary nerve in the quadrilateral space.

**Fig. 5:**
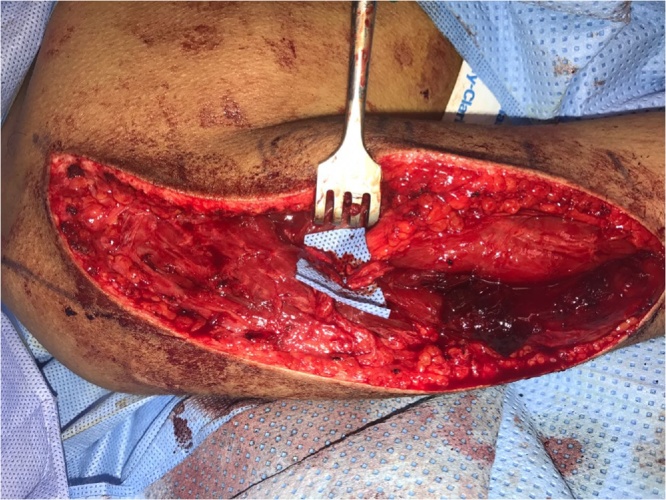
The divided axillary nerve and the divided triceps nerve are shown under the blue back-ground just prior to coaptation using fibrin glue.

The patient is positioned in the prone position. Localization of the axillary nerve and its cutaneous branch is done prior to prepping and draping. A longitudinal line is drawn from the postero-lateral acromion to the olecranon. A second transverse line is drawn from the axillary fold to intersect the first line at 90° angle. Anatomically, the quadrilateral space should be 2 cm cranial to this point of intersection [5]. This point is marked blue in Fig. 1a&b. Using 6–15 Mhz linear ultrasound probe (Fig. 1C) positioned at the postero-medial aspect of the upper arm in the sagittal plane, we first identify the humeral head and neck. The probe is then moved medially to view quadrilateral space and the target structures within the space (the circumflex artery and the axillary nerve which appears as hyper-echoic oval-shaped structure). The ultrasound probe is then moved to scan the cutaneous branch of the axillary nerve as it branches-off the main nerve trunk. Finally, the cutaneous branch is traced superficially till it becomes subcutaneous. In the current case, the cutaneous branch at the level of the skin is marked as a red circle in Fig. 2. The patient is now prepped and draped. A longitudinal surgical incision is made along the acromion-olecranon line passing through the previously located quadrilateral space point as well as the point of the cutaneous branch of the axillary nerve. The cutaneous branch is then identified and dissected (Fig. 3). Next, the cutaneous branch is followed retrograde to the axillary nerve in the quadrilateral space (Fig. 4). The motor part of the axillary nerve is isolated and divided as far as possible from its entry to the deltoid muscle. The radial nerve branches to the three heads of the triceps muscle are then identified. We usually use the motor branch to either the lateral or medial head of the triceps. The selected triceps motor branch is transected as distal as possible and is transposed proximally for coaptation to the divided deltoid nerve using fibrin glue (Fig. 5).

## 3 Discussion

Previous authors mentioned that the easiest way to identify the axillary nerve in the quadrilateral space is through retrograde dissection of the cutaneous branch of the nerve [2].

With ultrasound guidance, we found it is easier and more reliable to scan the main axillary nerve trunk in the quadrilateral space and then trace the cutaneous branch antegrade without ultrasound guidance, localization of the cutaneous branch of the nerve may be difficult especially in obese patients and in patients with previous surgery in the arm. Furthermore, residents-in-training usually take a long time localizing the cutaneous branch even in straight-forward cases. Hence, the above ultrasound-guided technique for pre-operative nerve localization is now preferred at our teaching institution.

Ultrasound guidance for localization of various nerves is now routinely done by anesthetists in the Operating Room. This localization is used for nerve blocks [6] and in patients with brachial plexus injuries [7]. Hence, the ultrasound machine and the expertise are already available in the operating room; and no special arrangements with the Radiology Department are needed. Finally, the marking and the ultrasound-guided localization described in our technique can be done in holding area in the awake patient and hence, no operative time is wasted. In fact, our technique saves operative room time because the localization point is accurate and the cutaneous branch is easily identified at the marked point once the skin incision is made.

## 4 Conclusion

A technique of identification of the cutaneous branch of the axillary nerve using anatomical landmarks and ultrasonography is described. The localization is accurate and is of help in patients undergoing triceps nerve-to-deltoid nerve transfer.

## Conflict of interest

None.

## Funding

None.

## Ethical approval

The study was approved by the research committee, National Hospital (Care), Riyadh, Saudi Arabia.

## Consent

Written informed consent was obtained from the patient for publication of this case report and accompanying images. A copy of the written consent is available for review by Editor-in-chief of this Journal on request.

## Author contribution

All authors contributed significantly and in agreement with the content of the manuscript. All authors participated in data collection and in writing of the manuscript.

## Registration of research studies

Not relevant here.

## Guarantor

M M Al-Qattan.

## Provenance and peer review

Not commissioned, externally peer reviewed.
